# Drug Use and Receipt of Highly Active Antiretroviral Therapy among HIV-Infected Persons in Two U.S. Clinic Cohorts

**DOI:** 10.1371/journal.pone.0018462

**Published:** 2011-04-25

**Authors:** Catherine C. McGowan, David D. Weinstein, Charles P. Samenow, Samuel E. Stinnette, Gema Barkanic, Peter F. Rebeiro, Timothy R. Sterling, Richard D. Moore, Todd Hulgan

**Affiliations:** 1 Division of Infectious Diseases, Department of Medicine, Vanderbilt University School of Medicine, Nashville, Tennessee, United States of America; 2 Department of Psychiatry, Vanderbilt University School of Medicine, Nashville, Tennessee, United States of America; 3 Center for Health Services Research, Vanderbilt University School of Medicine, Nashville, Tennessee, United States of America; 4 Division of Infectious Diseases, Johns Hopkins University School of Medicine, Baltimore, Maryland, United States of America; University of Sao Paulo, Brazil

## Abstract

**Objective:**

Drug use and receipt of highly active antiretroviral therapy (HAART) were assessed in HIV-infected persons from the Comprehensive Care Center (CCC; Nashville, TN) and Johns Hopkins University HIV Clinic (JHU; Baltimore, MD) between 1999 and 2005.

**Methods:**

Participants with and without injection drug use (IDU) history in the CCC and JHU cohorts were evaluated. Additional analysis of persons with history of IDU, non-injection drug use (NIDU), and no drug use from CCC were performed. Activity of IDU and NIDU also was assessed for the CCC cohort. HAART use and time on HAART were analyzed according to drug use category and site of care.

**Results:**

1745 persons were included from CCC: 268 (15%) with IDU history and 796 (46%) with NIDU history. 1977 persons were included from JHU: 731 (35%) with IDU history. Overall, the cohorts differed in IDU risk factor rates, age, race, sex, and time in follow-up. In multivariate analyses, IDU was associated with decreased HAART receipt overall (OR = 0.61, 95% CI: [0.45–0.84] and OR = 0.58, 95% CI: [0.46–0.73], respectively for CCC and JHU) and less time on HAART at JHU (0.70, [0.55–0.88]), but not statistically associated with time on HAART at CCC (0.78, [0.56–1.09]). NIDU was independently associated with decreased HAART receipt (0.62, [0.47–0.81]) and less time on HAART (0.66, [0.52–0.85]) at CCC. These associations were not altered significantly whether patients at CCC were categorized according to historical drug use or drug use during the study period.

**Conclusions:**

Persons with IDU history from both clinic populations were less likely to receive HAART and tended to have less cumulative time on HAART. Effects of NIDU were similar to IDU at CCC. NIDU without IDU is an important contributor to HAART utilization.

## Introduction

The dual global pandemics of substance abuse and HIV threaten not only individual but also public health. Injection drug use (IDU) was the HIV transmission risk factor for 35% of females and 20% of males with AIDS reported through 2007 in the United States (US) [1]. The role of IDU in HIV transmission varies according to geographic region. The distribution of AIDS cases due to IDU in 2006 was approximately 25% and 50% greater for females and males, respectively, in the Northeastern US compared with those in Southern and Midwestern states [2]. Beyond the US, IDU is fueling HIV transmission in Eastern Europe and Central, South, and South-East Asia, and is estimated to account for approximately one-third of new HIV infections outside Sub-Saharan Africa [3]. Injection drug users often receive their HIV diagnosis late and may have worse clinical outcomes compared to persons without IDU [4].

The use of highly active antiretroviral therapy (HAART) has led to sustained decreases in HIV-related morbidity and mortality [5], [6]. Successful long-term outcomes require a high degree of patient adherence and persistence with therapy [7], [8], [9]. Substance use may be associated with decreased adherence to HAART [10]–[12] and subsequent increased rates of virologic failure and HIV disease progression [13], [14]. However, the association of drug use with reduced adherence is not uniform, as participants in the Smartest Women's project reported relatively high levels of adherence regardless of current, former, and never drug use status [15]. Patient persistence with HAART, reflecting the duration of time on therapy, was decreased among former and current injection drug users in Baltimore, with 78% of individuals having one or more treatment interruptions; persons reporting daily IDU had a higher probability of treatment interruptions [16]. Whether non-injection drug use (NIDU) exerts a similar influence on patient persistence with HAART is not known [9].

Behaviors related to drug use and illicit drugs themselves may lead to poorer treatment outcomes and high risk of HIV transmission. Potential effects of drug use include neurocognitive impairment, psychosocial dysfunction, or exacerbation of psychiatric illness. Altered judgment and decision-making may lead to high-risk sexual behavior and attendant risk of acquiring other sexually-transmitted infections, which in turn may facilitate HIV transmission [17] and disease progression [18]. Exchange of sex for drugs or money may occur in users of any illicit substance, especially crack cocaine [19]–[21]. Persons with substance use disorders may transition through correctional facilities, which may be associated with cyclic interruptions in HIV treatment [9], [16]. There also are possible mechanisms by which cocaine and opiates could directly affect HIV disease outcomes. Studies *in vitro*, and in animals and humans have suggested that cocaine and opiates have direct effects on HIV replication and T cell immunology [22]–[27].

A history of IDU has been associated with delayed initiation of HAART [28]–[31]. Despite this, an increased risk of HIV disease progression and death has not been uniformly reported in studies of IDU in the HAART era [31]–[33]. These outcomes may vary due to differences in study design and the populations evaluated. NIDU also is common in patients with HIV infection; an estimated prevalence of 40–60% has been reported [34], [35]. Crack cocaine users have been shown to have worse HIV outcomes compared with non-users [36], and this effect was independent of HAART use in one cohort [37]. NIDU is difficult to characterize, however, as it often includes overlapping use of many substances, each with differing effects on the user. Patterns of NIDU over time may be heterogeneous, erratic, and dynamic, making accurate measurement of use over time complicated [38]. Few studies have examined the association of NIDU with HAART utilization and persistence. We conducted a retrospective study of two geographically distinct urban US cohorts to identify relationships between IDU, receipt of HAART, and demographic features of the cohorts. We also performed targeted analyses in a single cohort in the Southeastern US to characterize relationships between NIDU and HAART utilization in this setting.

## Methods

### Ethics Statement

The study was approved by the Vanderbilt University and Johns Hopkins Institutional Review Boards. Exemption from informed consent was approved by the Vanderbilt University Institutional Review Board. Written informed consent was obtained for all participants from Johns Hopkins. To maintain patient confidentiality, non-identifiable patient data were used in the analyses.

### Study Population and Database

The overall cohort included persons who had their first outpatient visit at the Vanderbilt-affiliated Comprehensive Care Center (CCC) in Nashville or the Moore Clinic for HIV Care at Johns Hopkins University (JHU) in Baltimore between January 1, 1999 and December 31, 2004. Subjects must have had at least two outpatient visits during the study period. Study follow-up for each patient was censored at the date of death, date of last clinic encounter if the patient met lost-to-follow-up criteria (defined as ≥18 months between clinic encounters without laboratory data during the intervening time), or December 31, 2005 if the last clinic encounter or death date was after that date.

The CCC is the largest HIV outpatient care center in Tennessee and includes three satellite clinics in rural Middle Tennessee. In addition to providing specialized HIV medical care, the CCC provides case management and coordination of mental health services including substance abuse treatment programs. Clinical data have been entered directly into an electronic medical record (EMR) since 1997, either by medical providers at the time of the patient encounter, by automated laboratory data upload, or by retrospective entry by clinic personnel (for updating data such as death, hospitalization, interim diagnoses, and laboratory results received from outside sources). Risk factors for HIV acquisition, including history of IDU, are identified by clinicians at patient enrollment at CCC. Medical charts were reviewed by trained abstractors to ascertain additional history of IDU, NIDU and heavy use or abuse of alcohol (defined below).

JHU provides integrated primary and subspecialty care for a large proportion of HIV-infected patients in the Baltimore metropolitan area. An observational, longitudinal, clinical cohort of adult patients receiving HIV care at JHU has been maintained since 1990 [39]. Enrollment into the cohort corresponds to enrollment at JHU. Comprehensive demographic, clinical, therapeutic, and laboratory data are collected at baseline (enrollment) and updated at 6-month intervals using structured data collection forms and coding criteria. These data are updated from clinic visits and inpatient admissions records, laboratory testing, pharmacy, social services, and all other available clinical sources via standardized abstraction. Nonexclusive risk factors for HIV acquisition (including IDU) are identified by clinicians when patients enroll in the cohort.

### Exposure and Outcome Definitions

Persons from the CCC cohort were categorized into three mutually exclusive groups for analysis: persons with an IDU history, persons with NIDU history, and persons with neither IDU nor NIDU history. The overall cohort (including JHU) was categorized into two groups for analysis: persons with an IDU history and those with no history of IDU. For the CCC cohort, persons were considered to have a history of IDU if there was any record of IDU as self-reported probable route of HIV infection, or any corresponding ICD-9 diagnoses or free text entered into the EMR by providers. For the JHU cohort, IDU as either provider-entered diagnosis or self-reported HIV risk is captured as part of structured data abstraction procedures [40].

NIDU at CCC was defined as any record of substance abuse/use, dependence, or addiction, or self-reported heavy alcohol use, or recreational drug use, of any kind or amount, excluding anyone whose HIV risk factor was IDU. After an electronic query of ICD-9 diagnosis codes for abuse and/or dependence identified persons with IDU as HIV risk factor and those with a history of NIDU, chart validation was performed on the remaining persons to assess substance use through notation by the provider of concern about drug or alcohol use, notation of a patient entering a drug or alcohol rehabilitation program, notation of non-injection prescription drug abuse, or indication of medical, legal, or social problems resulting from drug or alcohol use. Drugs used in the diagnosis of NIDU included cocaine, crack cocaine, methamphetamine, heroin, opiates, marijuana, and “party drugs” such as methylenedioxymethamphetamine and nitrous oxide. Alcohol use was categorized as NIDU if there was a provider notation in the chart of heavy alcohol use, or an alcohol-related clinical diagnosis (e.g. alcoholic liver disease). To account for potential weakness in the pertinence of a history of drug use to the receipt of HAART, additional reviews of charts of persons categorized as having historical IDU and NIDU were conducted at CCC to assess for active drug use during the study period.

Baseline (enrollment) demographics and laboratory values were obtained from the medical record. Baseline CD4^+^ T cell count and plasma HIV-1 RNA level were defined as the first values obtained ≤60 days prior to or any time after the first clinic visit between January 1, 1999 and December 31, 2004.

The primary outcomes of interest were ever receiving HAART and cumulative time on HAART during the study period. HAART was defined as (1) at least 2 nucleoside reverse-transcriptase inhibitors (NRTIs) in combination with at least 1 protease inhibitor (PI) and/or non-NRTI (NNRTI); (2) one NRTI and any PI and any NNRTI; or (3) at least 3 NRTIs. Persons who received HAART for ≥ 7 days were classified as having ever received HAART. Time on HAART included cumulative HAART between January 1, 1999 and December 31, 2005, including HAART that began prior to January 1, 1999. HAART start was set at January 1, 1999 if HAART start occurred prior to that date and HAART end date was set as December 31, 2005 if HAART stop occurred after that date. Proportional time on HAART was calculated as cumulative weeks on HAART for each individual who ever received HAART divided by the follow-up time in weeks for that individual. For analyses, this outcome was dichotomized as ≥ or <95% of available follow-up time on HAART [41].

### Statistical analysis

Categorical variables were compared with the χ^2^ or Fisher's exact tests. Continuous variables are shown as medians and were compared with the Wilcoxon rank-sum (Mann-Whitney *U*) or Kruskal-Wallis tests. Logistic regression models were constructed to analyze receipt of HAART and proportional time on HAART according to drug use category and site of care. Other covariates adjusted for in multivariate analyses included baseline demographics, cumulative follow-up time, CD4^+^ T cell count categorized as < or ≥200 cells/mm^3^, and HIV-1 RNA level categorized as ≤ or >5 log_10_ (100,000) copies/mL. Separate multivariate logistic regression models were constructed *a priori* for the CCC and JHU cohort databases. These included identical models without NIDU history (Model 1). A corresponding model to assess the effect of NIDU in the CCC cohort included the same demographic and laboratory variables as the other models as well as IDU and NIDU history (Model 2). Secondary models including active drug use during the study period, instead of historical drug use, were used to assess the association of activity of drug use with receipt of HAART and proportional time on HAART in the CCC cohort. All analyses were performed using Stata SE (version 10.0; Stata Corporation, College Station, TX).

## Results

### CCC cohort analysis, total and according to IDU risk and drug use categories (Table 1)

**Table 1:** Clinical and demographic characteristics of Comprehensive Care Center study cohort, total and according to IDU risk and drug use categories.^a^

Characteristic	Total cohort (n = 1745)	No IDU history			IDU history (n = 268)	P value^c^
		Total(n = 1477)	No drug use history (n = 681)	NIDU history^b^(n = 796)		
Age in years	37 (31–43)	37 (30–43)	36 (29–43)	37 (32–42)	41 (35–45)	<0.001
Black race	660 (38)	536 (37)	225 (33)	311 (39)	124 (46)	<0.001
Female sex	419 (24)	356 (24)	190 (28)	166 (21)	63 (24)	0.007
Follow-up time in weeks	119 (66–207)	124 (68–211)	122 (61–203)	125 (73–223)	100 (45–173)	<0.001
Baseline CD4^+^ T cell count^d^, cells/mm^3^	306 (136–504)	308 (136–500)	306 (120–483)	308 (151–513)	288 (140–506)	0.56
Baseline HIV-1 RNA^d^, log_10_ copies/mL	4.4 (3.2–5.0)	4.4 (3.3–5.1)	4.4 (3.1–5.1)	4.4 (3.4–5.1)	4.4 (3.0–4.9)	0.44
Baseline CD4^+^ T cell count^d^ <200 cells/mm^3^	582 (34)	492 (34)	227 (34)	265 (33)	90 (34)	0.99
Baseline HIV-1 RNA^d^ <400 copies/mL	299 (17)	247 (17)	136 (20)	111 (14)	52 (20)	0.004
HAART ever during study period^e^	1282 (73)	1109 (75)	536 (79)	573 (72)	173 (65)	<0.001
Time on HAART in weeks^f^	100 (46–175)	102 (48–177)	103 (46–175)	102 (53–179)	88 (30–162)	0.06
% follow-up time on HAART among ever exposed to HAART (n = 1282)	90 (58–99)	91 (58–99)	94 (61–99.8)	88 (54–98.7)	88 (61–99.5)	0.02

a Data are median (interquartile range) or n (%).

b Non-injection drug use history defined as any record of substance abuse/dependence/addiction or self-reported use of heavy alcohol or past or current recreational drug use, of any kind or amount, excluding anyone whose HIV risk is injection drug use.

c P values are for comparisons between the three drug use categories using Kruskal-Wallis or Chi-squared tests.

d CD4^+^ T cell counts and plasma HIV-1 RNA levels not available for all persons; data shown are for those persons with data available. Baseline laboratory values defined as the first value obtained on or after the initial office visit, between January 1, 1999 and December 31, 2004.

e Defined as at least 7 days of continuous HAART.

f Among persons ever exposed to HAART.

IDU  =  injection drug use; CCC  =  Comprehensive Care Center, Nashville, TN; HAART  =  highly active antiretroviral therapy.

The total CCC study cohort was predominantly white and male and median age was 37 years. Of the 1745 persons included, 796 (46%) persons met criteria for history of NIDU, 268 (15%) persons met criteria for history of IDU, and 681 (39%) persons had no drug use history. Comparisons between the three drug use categories demonstrated statistically significant differences in age, race, sex, and follow-up time during the study period. There were differences in likelihood of ever receiving HAART, with persons with no drug use history having the greatest likelihood and persons with a history of IDU having the lowest. The proportion of follow-up time on HAART was also greatest among those with no drug use compared with those having a history of either NIDU or IDU. There was no significant difference in median CD4^+^ T cell count or plasma HIV-1 RNA level at baseline between the substance use categories. The median year of cohort entry was 2002. Earlier year of cohort entry at CCC was associated with greater likelihood of ever receiving HAART, but lower likelihood of having ≥95% of follow-up time on HAART (P<0.01 for both).

There were 76 participants with active IDU and 776 with active NIDU recorded during the study period. Active NIDU was present for 644 persons (81%) who were categorized as history of NIDU and for 132 persons (49%) categorized as history of IDU. Among individuals with history of IDU, 69 (26%) had active IDU during the study period.

### JHU cohort analysis, total and according to IDU status (Table 2)

**Table 2:** Clinical and demographic characteristics of the Johns Hopkins University study cohort, total and according to IDU risk category.^a^

Characteristic	Total cohort (n = 1977)	No IDU history (n = 1246)	IDU history (n = 731)	P value^b^
Age in years	39 (34–45)	38 (32–44)	41 (37–46)	<0.001
Black race	1458 (74)	859 (69)	599 (82)	<0.001
Female sex	683 (35)	438 (35)	245 (34)	0.46
Follow-up time in weeks	171 (93–255)	176 (97–257)	164 (88–162)	0.25
Baseline CD4^+^ T cell count^c^, cells/mm^3^	273 (105–466)	274 (98–467)	272 (117–461)	0.79
Baseline HIV-1 RNA^c^, log_10_ copies/mL	4.4 (3.1–5.1)	4.4 (3.1–5.1)	4.4 (3.2–5.1)	0.56
Baseline CD4^+^ T cell count^c^ <200 cells/mm^3^	764 (39)	486 (39)	278 (38)	0.70
Baseline HIV-1 RNA^c^ <400 copies/mL	352 (18)	230 (19)	122 (17)	0.60
HAART ever during study period^d^	1429 (72)	948 (76)	481 (66)	<0.001
Time on HAART in weeks^e^	123 (52–220)	129 (57–226)	112 (41–209)	<0.001
% follow-up time on HAART among ever exposed to HAART (n = 1429)	96 (55–100)	97 (63–100)	87 (40–100)	<0.001

a Data are median (interquartile range) or n (%).

b P-values are for comparisons between IDU and no IDU history groups using Wilcoxon rank-sum or Fisher's exact tests.

c CD4^+^ T cell counts and plasma HIV-1 RNA levels not available for all persons; data shown are for those persons with data available. Baseline laboratory values defined as the first value obtained on or after the initial office visit, between January 1, 1999 and December 31, 2004.

d Defined as at least 7 days of continuous HAART.

e Among persons ever exposed to HAART.

IDU  =  injection drug use; HAART  =  highly active antiretroviral therapy.

The total JHU study cohort was predominantly black and male and median age was 39 years. Of the 1977 persons included, 731 (37%) persons met criteria for history of IDU. Comparisons between IDU and non-IDU categories demonstrated that individuals with a history of IDU were significantly older, more likely to be black, and less likely to ever receive HAART, and had less follow-up time on HAART during the study period. There was no significant difference in sex, follow-up time during the study period and median CD4^+^ T cell count and HIV-1 RNA level at baseline between the drug use categories. The median year of cohort entry was 2001. Earlier year of cohort entry at JHU was associated with greater likelihood of ever receiving HAART (P = 0.04) but not associated with having ≥95% of follow-up time on HAART (P = 0.5).

### Multivariate models of predictors of ever receiving HAART and receiving HAART ≥95% of follow-up time in the CCC cohort (Table 3)

Two logistic regression models were used to assess the effects of age, baseline laboratory values, sex, race, follow-up time, and drug use history on the receipt of HAART in the CCC study cohort. Model 1included only IDU history to be consistent with results of the corresponding model of JHU data (shown in Table 4). Model 2 included both IDU and NIDU history. CCC participants with baseline CD4^+^ T cell count <200 cells/mm^3^ and HIV-1 RNA level >5 log_10_ copies/mL and longer follow-up time on study were significantly more likely to receive HAART, whereas black race, IDU history and NIDU history each were associated with a lower likelihood of HAART receipt. Among persons exposed to HAART, baseline HIV-1 RNA level >5 log_10_ copies/mL, black race, and NIDU history were inversely associated with receipt of HAART ≥95% of follow-up time. In Model 2 adjusted for both NIDU and IDU, history of IDU was significantly associated with a decreased likelihood of receipt of HAART ≥95% of follow-up time. Although female sex had a positive association with HAART use ever, women were significantly less likely to receive HAART ≥95% of follow-up time in the CCC cohort.

**Table 3:** Multivariate logistic regression models of predictors of (A) ever receiving HAART and (B) receiving HAART ≥95% of follow-up time in the Comprehensive Care Center study cohort.

	A. Predictors of ever receiving HAART (N = 1730)						B. Predictors of receiving HAART ≥95% of follow-up time^a^ (N = 1275)					
Covariate	Model 1^b^			Model 2^b^			Model 1 b			Model 2 b		
	OR	95% CI	P	OR	95% CI	P	OR	95% CI	P	OR	95% CI	P
Age (per year)	1.03	1.02–1.04	<0.001	1.03	1.02–1.04	<0.001	1.02	1.01–1.04	<0.001	1.02	1.01–1.04	0.001
Baseline CD4^+^ T cell count^b^ <200 cells/mm^3^	4.40	3.17–6.12	<0.001	4.43	3.19–6.16	<0.001	1.13	0.88–1.46	0.33	1.15	0.89–1.48	0.29
Baseline HIV-1 RNA^c^ >5 log_10_ copies/mL	1.67	1.12–2.34	0.003	1.68	1.20–2.35	0.003	0.63	0.48–0.82	0.001	0.62	0.47–0.81	0.001
Female sex	1.27	0.96 1.69	0.10	1.22	0.92–1.63	0.17	0.75	0.57–0.99	0.05	0.71	0.53–0.94	0.02
Black race	0.56	0.43–0.71	<0.001	0.57	0.44–0.73	<0.001	0.64	0.50–0.81	<0.001	0.65	0.51–0.83	0.001
Follow-up time (per year)	1.66	1.53–1.81	<0.001	1.68	1.54–1.83	<0.001	0.96	0.90–1.02	0.21	0.97	0.91–1.03	0.28
IDU history	0.61	0.45–0.84	0.003	0.47	0.33–0.67	<0.001	0.78	0.56–1.09	0.15	0.63	0.44–0.90	0.01
NIDU history b	-	-	-	0.62	0.47–0.81	0.001	-	-	-	0.66	0.52–0.85	0.001

a Among those ever exposed to HAART.

b Model 1 did not include NIDU history, in order to be consistent with results of the corresponding model of JHU data. Model 2 included both IDU and NIDU, as well as the other covariates shown. IDU and NIDU were mutually exclusive definitions (see METHODS), thus Model 2 results do not denote independent relationships between drug-use categories and outcome variables.

c CD4^+^ T cell counts and plasma HIV-1 RNA levels not available for all persons; data shown are for those persons with data available. Baseline laboratory values are defined as the first values obtained on or after the initial office visit, between January 1, 1999 and December 31, 2004.

HAART  =  highly active antiretroviral therapy; OR  =  odds ratio; CI  =  confidence interval; IDU  =  injection drug use; NIDU  =  non-injection drug use.

**Table 4:** Multivariate logistic regression models of predictors of (A) ever receiving HAART and (B) receiving HAART ≥95% of follow-up time in the Johns Hopkins University study cohort.

Covariate	Predictors of ever receiving HAART (N = 1960)			Predictors of receiving HAART ≥95% of follow-up time^a^ (N = 1422)		
	OR	95% CI	P	OR	95% CI	P
Age (per year)	1.01	0.999–1.02	0.08	1.02	1.00–1.03	0.02
Baseline CD4^+^ T cell count^b^ <200 cells/mm^3^	5.16	3.90–6.82	<0.001	1.82	1.45–2.29	<0.001
Baseline HIV-1 RNA^b^ >5 log _10_ copies/mL	1.93	1.44–2.59	<0.001	0.76	0.60–0.98	0.03
Female sex	0.71	0.56–0.89	0.003	0.84	0.67–1.06	0.15
Black race	0.70	0.54–0.91	0.007	0.62	0.48–0.79	<0.001
Follow-up time (per year)	1.32	1.24–1.40	<0.001	1.12	1.06–1.19	<0.001
IDU history	0.58	0.46–0.73	<0.001	0.70	0.55–0.88	0.002

a Among those ever exposed to HAART.

b CD4^+^ T cell counts and plasma HIV-1 RNA levels not available for all persons; data shown are for those persons with data available. Baseline laboratory values are defined as the first values obtained on or after the initial office visit, between January 1, 1999 and December 31, 2004.

HAART  =  highly active antiretroviral therapy; OR  =  odds ratio; CI  =  confidence interval; IDU  =  injection drug use.

### Multivariate models of predictors of ever receiving HAART and receiving HAART ≥95% of follow-up time in the JHU cohort (Table 4)

Receipt of HAART in the JHU study cohort was assessed using logistic regression models that included the effects of age, baseline laboratory values, sex, race, follow-up time, and IDU history. JHU participants with baseline CD4^+^ T cell count <200 cells/mm^3^ and HIV-1 RNA level >5 log_10_ copies/mL and longer follow-up time on study were significantly more likely to receive HAART, whereas female sex, black race, and IDU history each were associated with a lower likelihood of HAART receipt. Among persons receiving HAART, baseline CD4^+^ T cell count <200 cells/mm^3^ and longer follow-up time on study were associated with receipt of HAART ≥95% of total follow-up time. Baseline HIV-1 RNA level >5 log_10_ copies/mL, black race, and IDU history were inversely associated with receipt of HAART ≥95% of total follow-up time.

### CCC cohort analysis using multivariate models of predictors of receiving HAART, accounting for active drug use during the study period

Models including active drug use during the study period, instead of historical drug use, were used to assess the association of activity of drug use with receipt of HAART and proportional time on HAART in the CCC cohort. These analyses showed patterns of associations between active drug use and receipt of HAART that were similar to those observed using historical drug use. In models adjusted for both active NIDU and IDU, IDU during the study period was associated with decreased likelihood of receipt of HAART ever (OR = 0.51, 95% CI: 0.29 to 0.89) and ≥95% of total follow-up time (OR = 0.46, 95% CI: 0.24 to 0.87), as was NIDU (OR = 0.60, 95% CI: 0.47 to 0.77 and OR = 0.72, 95% CI: 0.57 to 0.91, respectively for HAART ever and ≥95% of total follow-up time).

## Discussion

This study was based on data compiled from the CCC, which serves urban and rural Tennessee, and the urban JHU cohort in Baltimore, Maryland, permitting analysis of IDU and its effects on HAART utilization in two different HIV outpatient populations with up to five years of follow-up. The large sample size provides an opportunity to add to the current literature that reports inconsistent effects of IDU on various HIV outcomes, including HAART use [35]. Associations between NIDU and HIV disease progression and mortality [36], [37], and current HAART use [42], recently have been reported. However, our study provides important new information from a large cohort on NIDU and HAART use, both ever and over time. Given that persons with a history of IDU and NIDU constitute a large proportion of HIV-infected individuals, clarifying the effects of drug use on HAART utilization in this subpopulation is important.

The current study showed that a history of IDU was associated with a decreased likelihood of ever receiving HAART after presentation for care. The lower likelihood of those with IDU history remaining on HAART at JHU may reflect a higher prevalence of active IDU in Baltimore compared to Nashville [43]. Certain factors related to ongoing IDU such as poor nutrition, coexistent mental health disorders, hepatitis co-infection, and weaker immune response, may limit the ability of persons with IDU to tolerate or adhere to HAART [35], [44].

For this analysis, NIDU history was assessed only in the CCC cohort and was also associated with a lower likelihood of ever receiving HAART as well as a decreased likelihood of remaining on HAART ≥95% of the total follow-up time. NIDU affected HAART utilization after adjusting for race, sex, follow-up time, and baseline HIV-1 RNA and CD4^+^ T cell count. To our knowledge, this is the first analysis to assess NIDU and HAART utilization in an HIV-infected population. The mechanisms by which NIDU influences HAART utilization and exposure are presumably similar to those for IDU [35], though the greater heterogeneity of illicit substances included in our NIDU definition make the magnitude of the observed association somewhat surprising. Future studies should examine the specific influence of individual NIDU substances and of polysubstance abuse, categories that were not determined for this analysis.

An unexpected finding was that sex was associated with ever receiving HAART as well as the probability of remaining on HAART during the study period, but the direction of association differed according to site of care. Females were more likely to receive HAART at CCC, but were less likely to remain on HAART when compared with men. At JHU, women were less likely to receive any HAART, but once on therapy, sex was not associated with likelihood of remaining on HAART. In a Canadian cohort, women were significantly less likely to begin HAART compared with men despite equal access to care [45], and women were significantly less likely than men to be on HAART in a US multi-center study [42]. Rates of HAART discontinuation did not vary according to sex in a cohort from the Southeastern US, but reasons for discontinuation did; women were more likely to interrupt therapy due to non-adherence, to report unique patterns of adverse events, and to spend more cumulative time off HAART compared to men [46]. A recent analysis of participants in the Women's Interagency HIV Study and the Multicenter AIDS Cohort Study found that older age and Caucasian race were associated with a higher prevalence of HAART-related symptoms, particularly among women; however, depression and a prior diagnosis of AIDS were the strongest predictors of symptoms in both cohorts [47]. Factors that influence the decision to begin HAART may differ from those that affect the ability to adhere to therapy. We speculate that the differential associations observed in the present study are likely due to unmeasured factors among females that differed between sites, including cohort heterogeneity [48]. These might include activity of substance use (especially IDU), pregnancy rates, co-morbidities such as hepatitis C infection and mental health diagnoses, general clinic practice patterns, and socioeconomic and housing statuses.

One of the strengths of this study, the ability to compare across two geographically distinct urban cohorts, is also a potential weakness. Differences in the clinic populations are evidenced by IDU risk factor rates, baseline demographics such as age, race, and sex, and time in follow-up. Among persons with an IDU risk history, those in the JHU cohort were more likely to be female and of black race than those in the CCC cohort, but the cohorts did not differ significantly with respect to baseline HIV-1 RNA or overall rates of HAART receipt. There also were differences in ascertainment of drug use at CCC compared with JHU. Persons classified solely on the basis of IDU history in both cohorts may have injected heroin or stimulants, such as cocaine and methamphetamine, and may also have used drugs through other routes. Non-injection drug use was characterized only for the CCC, and in this cohort half of those persons categorized with historical IDU had active NIDU during the study period. However, specific categories of NIDU were not distinguished in the CCC analysis. Stimulant users are reported to have the greatest risk of poor adherence compared with both non-drug-users and non-stimulant drug users [11].

Methodological differences in data collection and different ascertainment of behavioral information between CCC and JHU may also have affected our findings. Medical chart review and data abstraction were performed for CCC patients, whereas history of IDU was determined for JHU patients on the basis of reported risk factor for HIV acquisition. Documentation of substance use during routine patient care could be affected by provider reliability or individual presumptions. Also, substance-using individuals may minimize the frequency and quantity of drug use depending on the circumstances of self-report (e.g. personal interview versus anonymous survey), which were not standardized for either cohort.

Bias is inherent in observational data of this nature, and baseline status may have differed among subjects. For example, not all patients were HAART-naïve, some were on HAART, and others may have initiated therapy later in the study period. Analyses limited to persons whose baseline CD4^+^ T cell count was ≤200 were performed in the CCC cohort to minimize these biases, as all such persons would meet eligibility for HAART initiation. IDU history remained strongly associated with decreased likelihood of receipt of HAART in this model. Adherence to HAART was not assessed for either cohort in this retrospective analysis. Although we determined cumulative HAART exposure, we could not account for unrecorded drug “holidays” or unreported treatment interruptions that were relatively common in practice at times during the study period [49].

Substance abuse is by nature a dynamic and chronic process, and this variability may explain discordant findings in other studies [32], [38], [50]–[52]. In a prior report of IDU in Baltimore, only approximately 30% of individuals persistently used drugs during an average follow-up of 8 years [53]. Switching from substance use to non-use is strongly associated with improvements in antiretroviral therapy adherence and HIV treatment outcomes compared with ongoing use [14], [38], [54]. Additionally, active substance users experience extraordinary instability and are frequently transitioning residences, living on the street, or facing cyclic incarceration, which may lead to nonpersistence with HIV therapy [16], [55].

The present study demonstrated that for the CCC cohort, associations between drug use, receipt of HAART, and proportional time on HAART were not altered significantly whether patients were categorized according to historical drug use or drug use during the study period, which may have occurred before, during or after HAART use. This contrasts with findings from previous studies. For example, a prospective observational study found that historic IDU or any illicit drug history were not associated with decreased patient adherence to HAART, whereas recent drug activity was associated with poorer adherence [10]. The association of decreased receipt of HAART in both persons with historic drug use and active drug users may reflect patient-related factors or, alternatively, characteristics of care providers and the systems within which they work. After controlling for numerous other covariates, Ding et al. found that persons with IDU who were treated by physicians with negative attitudes towards IDU were half as likely to have been exposed to HAART compared to persons with non-IDU, or those treated by physicians with positive attitudes [56]. In a multisite cohort of active injection drug users, HAART use was independently predicted by better patient-provider engagement and stable housing [57].

HIV infection and substance abuse are highly interrelated chronic diseases with consequences that are expanding worldwide. This study demonstrates important negative associations between IDU, NIDU and HAART utilization. Future studies should better characterize patient-level, provider-level, and structural factors that may contribute to utilization of HAART in drug users, and further clarify the effects of different types of drug use on HAART utilization and HIV disease progression.

## References

[1] Centers for Disease Control and Prevention. HIV/AIDS Surveillance Report, 2007. Vol. 19. Atlanta: U.S. Department of Health and Human Services, Centers for Disease Control and Prevention (2009). CDC website. Available: http://www.cdc.gov/hiv/surveillance/resources/reports/2007report/pdf/cover.pdf. Accessed 1 April 2011

[2] Centers for Disease Control and Prevention. Cases of HIV infection and AIDS in urban and rural areas of the United States, 2006. HIV/AIDS Surveillance Supplemental Report (2008). CDC website. Available: http://www.cdc.gov/hiv/topics/surveillance/resources/reports/2008supp_vol13no2/pdf/HIVAIDS_SSR_Vol13_No2.pdf. Accessed 1 April 2011

[3] UNAIDS 2008 Report on the global AIDS epidemic. UNAIDS website. Available: http://www.unaids.org/en/KnowledgeCentre/HIVData/GlobalReport/2008/. Accessed 1 April 2011

[4] Grigoryan A, Hall HI, Durant T, Wei X (2009). Late HIV diagnosis and determinants of progression to AIDS or death after HIV diagnosis among injection drug users, 33 US States, 1996-2004.. PLoS One.

[5] Palella FJ, Delaney KM, Moorman AC, Loveless MO, Fuhrer J (1998). Declining morbidity and mortality among patients with advanced human immunodeficiency virus infection. HIV Outpatient Study Investigators.. N Engl J Med.

[6] Mocroft A, Ledergerber B, Katlama C, Kirk O, Reiss P (2003). Decline in the AIDS and death rates in the EuroSIDA study: an observational study.. Lancet.

[7] Hogg RS, Heath K, Bangsberg D, Yip B, Press N (2002). Intermittent use of triple-combination therapy is predictive of mortality at baseline and after 1 year of follow-up.. AIDS.

[8] Lucas GM, Chaisson RE, Moore RD (2003). Survival in an urban HIV-1 clinic in the era of highly active antiretroviral therapy: a 5-year cohort study.. J Acquir Immune Defic Syndr.

[9] Bae JW, Guyer W, Grimm K, Altice FL (2011). Medication persistence in the treatment of HIV infection: a review of the literature and implications for future clinical care and research.. AIDS.

[10] Golin CE, Liu H, Hays RD, Miller LG, Beck CK (2002). A prospective study of predictors of adherence to combination antiretroviral medication.. J Gen Intern Med.

[11] Hinkin CH, Barclay TR, Castellon SA, Levine AJ, Durvasula RS (2007). Drug use and medication adherence among HIV-1 infected individuals.. AIDS Behav.

[12] Tucker JS, Burnam MA, Sherbourne CD, Kung FY, Gifford AL (2003). Substance use and mental health correlates of nonadherence to antiretroviral medications in a sample of patients with human immunodeficiency virus infection.. Am J Med.

[13] Lucas GM, Cheever LW, Chaisson RE, Moore RD (2001). Detrimental effects of continued illicit drug use on the treatment of HIV-1 infection.. J Acquir Immune Defic Syndr.

[14] Arnsten JH, Demas PA, Grant RW, Gourevitch MN, Farzadegan H (2002). Impact of active drug use on antiretroviral therapy adherence and viral suppression in HIV-infected drug users.. J Gen Intern Med.

[15] Lopez E, Jones DL, Ishii M, Tobin JN, Weiss SM (2007). HIV medication adherence and substance use: The Smartest Women's Project.. Am J Infect Dis.

[16] Kavasery R, Galai N, Astemborski J, Lucas GM, Celentano DD (2009). Nonstructured treatment interruptions among injection drug users in Baltimore, MD.. J Acquir Immune Defic Syndr.

[17] Freeman EE, Weiss HA, Glynn JR, Cross PL, Whitworth JA (2006). Herpes simplex virus 2 infection increases HIV acquisition in men and women: systematic review and meta-analysis of longitudinal studies.. AIDS.

[18] Baeten JM, Strick LB, Lucchetti A, Whittington WL, Sanchez J (2008). Herpes simplex virus (HSV)-suppressive therapy decreases plasma and genital HIV-1 levels in HSV-2/HIV-1 coinfected women: a randomized, placebo-controlled, cross-over trial.. J Infect Dis.

[19] Booth RE, Watters JK, Chitwood DD (1993). HIV risk-related sex behaviors among injection drug users, crack smokers, and injection drug users who smoke crack.. Am J Public Health.

[20] Strathdee SA, Sherman SG (2003). The role of sexual transmission of HIV infection among injection and non-injection drug users.. J Urban Health.

[21] Cavazos-Rehg PA, Spitznagel EL, Schootman M, Strickland JR, Afful SE (2009). Risky sexual behaviors and sexually transmitted diseases: a comparison study of cocaine-dependent individuals in treatment versus a community-matched sample.. AIDS Patient Care STDS.

[22] Bryant HU, Bernton EW, Holaday JW (1988). Morphine pellet-induced immunomodulation in mice: temporal relationships.. J Pharmacol Exp Ther.

[23] Carr DJ, Gebhardt BM, Paul D (1993). Alpha adrenergic and mu-2 opioid receptors are involved in morphine-induced suppression of splenocyte natural killer activity.. J Pharmacol Exp Ther.

[24] Baldwin GC, Roth MD, Tashkin DP (1998). Acute and chronic effects of cocaine on the immune system and the possible link to AIDS.. J Neuroimmunol.

[25] Xu W, Flick T, Mitchel J, Knowles C, Ault K (1999). Cocaine effects on immunocompetent cells: an observation of in vitro cocaine exposure.. Int J Immunopharmacol.

[26] Roth MD, Tashkin DP, Choi R, Jamieson BD, Zack JA (2002). Cocaine enhances human immunodeficiency virus replication in a model of severe combined immunodeficient mice implanted with human peripheral blood leukocytes.. J Infect Dis.

[27] Cabral GA (2006). Drugs of abuse, immune modulation, and AIDS.. J Neuroimmune Pharmacol.

[28] Strathdee SA, Palepu A, Cornelisse PG, Yip B, O'Shaughnessy MV (1998). Barriers to use of free antiretroviral therapy in injection drug users.. JAMA.

[29] Murri R, Fantoni M, Del Borgo C, Izzi I, Visona R (1999). Intravenous drug use, relationship with providers, and stage of HIV disease influence the prescription rates of protease inhibitors.. J Acquir Immune Defic Syndr.

[30] Celentano DD, Galai N, Sethi AK, Shah NG, Strathdee SA (2001). Time to initiating highly active antiretroviral therapy among HIV-infected injection drug users.. AIDS.

[31] Rodriguez-Arenas MA, Jarrin I, Del Amo J, Iribarren JA, Moreno S (2006). Delay in the initiation of HAART, poorer virological response, and higher mortality among HIV-infected injecting drug users in Spain.. AIDS Res Hum Retroviruses.

[32] Poundstone KE, Chaisson RE, Moore RD (2001). Differences in HIV disease progression by injection drug use and by sex in the era of highly active antiretroviral therapy.. AIDS.

[33] Moore RD, Keruly JC, Chaisson RE (2004). Differences in HIV disease progression by injecting drug use in HIV-infected persons in care.. J Acquir Immune Defic Syndr.

[34] Galvan FH, Bing EG, Fleishman JA, London AS, Caetano R (2002). The prevalence of alcohol consumption and heavy drinking among people with HIV in the United States: results from the HIV Cost and Services Utilization Study.. J Stud Alcohol.

[35] Chander G, Himelhoch S, Moore RD (2006). Substance abuse and psychiatric disorders in HIV-positive patients: epidemiology and impact on antiretroviral therapy.. Drugs.

[36] Cook JA, Burke-Miller JK, Cohen MH, Cook RL, Vlahov D (2008). Crack cocaine, disease progression, and mortality in a multicenter cohort of HIV-1 positive women.. AIDS.

[37] Baum MK, Rafie C, Lai S, Sales S, Page B (2009). Crack-cocaine use accelerates HIV disease progression in a cohort of HIV-positive drug users.. J Acquir Immune Defic Syndr.

[38] Lucas GM, Griswold M, Gebo KA, Keruly J, Chaisson RE (2006). Illicit drug use and HIV-1 disease progression: a longitudinal study in the era of highly active antiretroviral therapy.. Am J Epidemiol.

[39] Chaisson RE, Keruly JC, Moore RD (1995). Race, sex, drug use, and progression of human immunodeficiency virus disease.. N Engl J Med.

[40] Moore RD (1998). Understanding the clinical and economic outcomes of HIV therapy: the Johns Hopkins HIV clinical practice cohort.. J Acquir Immune Defic Syndr Hum Retrovirol.

[41] Paterson DL, Swindells S, Mohr J, Brester M, Vergis EN (2000). Adherence to protease inhibitor therapy and outcomes in patients with HIV infection.. Ann Intern Med.

[42] Cofrancesco J, Scherzer R, Tien PC, Gibert CL, Southwell H (2008). Illicit drug use and HIV treatment outcomes in a US cohort.. AIDS.

[43] National Institute on Drug Abuse. Community Epidemiology Work Group. Epidemiologic Trends in Drug Abuse, 2006. NIDA website. Available at: http://www.drugabuse.gov/PDF/CEWG/Vol1_106.pdf. Accessed 1 April 2011

[44] Braitstein P, Justice A, Bangsberg DR, Yip B, Alfonso V (2006). Hepatitis C coinfection is independently associated with decreased adherence to antiretroviral therapy in a population-based HIV cohort.. AIDS.

[45] Mocroft A, Gill MJ, Davidson W, Phillips AN (2000). Are there gender differences in starting protease inhibitors, HAART, and disease progression despite equal access to care?. J Acquir Immune Defic Syndr.

[46] Kempf MC, Pisu M, Dumcheva A, Westfall AO, Kilby JM (2009). Gender Differences in Discontinuation of Antiretroviral Treatment Regimens.. J Acquir Immune Defic Syndr.

[47] Silverberg MJ, Jacobson LP, French AL, Witt MD, Gange SJ (2009). Age and racial/ethnic differences in the prevalence of reported symptoms in human immunodeficiency virus-infected persons on antiretroviral therapy.. J Pain Symptom Manage.

[48] Shepherd BE, Sterling TR, Moore RD, Raffanti SP, Hulgan T (2009). Cross-cohort heterogeneity encountered while validating a model for HIV disease progression among antiretroviral initiators.. J Clin Epidemiol.

[49] Lori F, Lisziewicz J (2001). Structured treatment interruptions for the management of HIV infection.. JAMA.

[50] Junghans C, Low N, Chan P, Witschi A, Vernazza P (1999). Uniform risk of clinical progression despite differences in utilization of highly active antiretroviral therapy: Swiss HIV Cohort Study.. AIDS.

[51] Mocroft A, Madge S, Johnson AM, Lazzarin A, Clumeck N (1999). A comparison of exposure groups in the EuroSIDA study: starting highly active antiretroviral therapy (HAART), response to HAART, and survival.. J Acquir Immune Defic Syndr.

[52] Sackoff JE, Hanna DB, Pfeiffer MR, Torian LV (2006). Causes of death among persons with AIDS in the era of highly active antiretroviral therapy: New York City.. Ann Intern Med.

[53] Galai N, Safaeian M, Vlahov D, Bolotin A, Celentano DD (2003). Longitudinal patterns of drug injection behavior in the ALIVE Study cohort,1988-2000: description and determinants.. Am J Epidemiol.

[54] Palepu A, Tyndall M, Yip B, O'Shaughnessy MV, Hogg RS (2003). Impaired virologic response to highly active antiretroviral therapy associated with ongoing injection drug use.. J Acquir Immune Defic Syndr.

[55] Mitty JA, Macalino GE, Bazerman LB, Loewenthal HG, Hogan JW (2005). The use of community-based modified directly observed therapy for the treatment of HIV-infected persons.. J Acquir Immune Defic Syndr.

[56] Ding L, Landon BE, Wilson IB, Wong MD, Shapiro MF (2005). Predictors and consequences of negative physician attitudes toward HIV-infected injection drug users.. Arch Intern Med.

[57] Knowlton AR, Arnsten JH, Eldred LJ, Wilkinson JD, Shade SB (2010). Antiretroviral use among active injection-drug users: the role of patient-provider engagement and structural factors.. AIDS Patient Care STDs.

